# pH Gradient-Driven Loading of Doxorubicin into Niosomes: A Comparative Study Using Bromocresol Green as a Visual Indicator

**DOI:** 10.3390/pharmaceutics17070862

**Published:** 2025-06-30

**Authors:** Mohammed Altaee, Ahmed Mostafa Faheem, Amal Ali Elkordy

**Affiliations:** 1 School of Pharmacy and Pharmaceutical Sciences, Faculty of Health Sciences and Wellbeing, University of Sunderland, Sunderland SR1 3SD, UK; bh06kp@student.sunderland.ac.uk (M.A.); ahmed.faheem@sunderland.ac.uk (A.M.F.); 2 RAK Medical and Health Sciences University, Ras Al Khaimah P.O. Box 11172, United Arab Emirates

**Keywords:** niosomes, pH indicator, bromocresol green, doxorubicin, Span 60, Solutol HS-15, active loading, remote loading

## Abstract

**Background**: The active (remote) loading of drugs into nanoparticulate systems via the pH gradient technique has been proven highly successful in liposomes, as numerous formulations have reached the market. However, this is not the case for niosomes, as the full potential of this area remains largely undiscovered. The purpose of this research is to study the effect of different co-surfactants (Cremophor RH 40, Cremophor ELP and Solutol HS-15) on stabilising the niosomal membrane to enable the creation of a pH gradient. **Methods**: For visualisation of pH gradients, pH indicator bromocresol green (BCG) was used as a novel encapsulated model molecule to visually investigate the ability of niosomes to entrap drugs through active loading. Thereafter, the optimised BCG niosomal formulation was applied to encapsulate a therapeutic drug molecule, doxorubicin, via pH gradient active loading. Niosomes were formulated via thin-film hydration using Span 60, cholesterol, with or without co-surfactants. Thin films were hydrated with either Trizma buffer or HEPES buffer for BCG, or ammonium sulfate for doxorubicin. The niosomes’ outer membrane pH was adjusted via either the addition of HCl or citric acid in the case of BCG, or by passing the niosomes through a Sephadex G50 gel column, pre-equilibrated with PBS or Trizma buffer, in the case of doxorubicin. **Results**: Niosomes formulated with Span 60 and cholesterol could not be formed at acidic pH and thus could not create a pH gradient. All three co-surfactants, when added to Span 60 and cholesterol, stabilised the niosomes and enabled them to form a pH gradient. Niosomes (after size reduction) containing Solutol HS-15 showed significantly higher entrapment efficiency of BCG when compared to Cremophor RH 40 and Cremophor ELP (67.86% vs. 15.57% vs. 17.81%, respectively, with sizes of 159.6 nm, 177.9 nm and 219.1 nm, respectively). The use of HEPES buffer resulted in a higher EE of BCG compared to Trizma buffer (72.85% vs. 67.86%) and achieved a size of 283.4 nm. The Solutol HS-15 containing formulation has exhibited 68.28% EE of doxorubicin with ammonium sulfate as the inner buffer, while the external buffer was Trizma with a size of 241.1 nm after extrusion. **Conclusions**: Niosomal formulations containing Solutol HS-15 are highly promising for remote drug loading. The novel use of BCG for studying pH gradient and drug loading into niosomes has proved beneficial and successful.

## 1. Introduction

**Figure 1:**
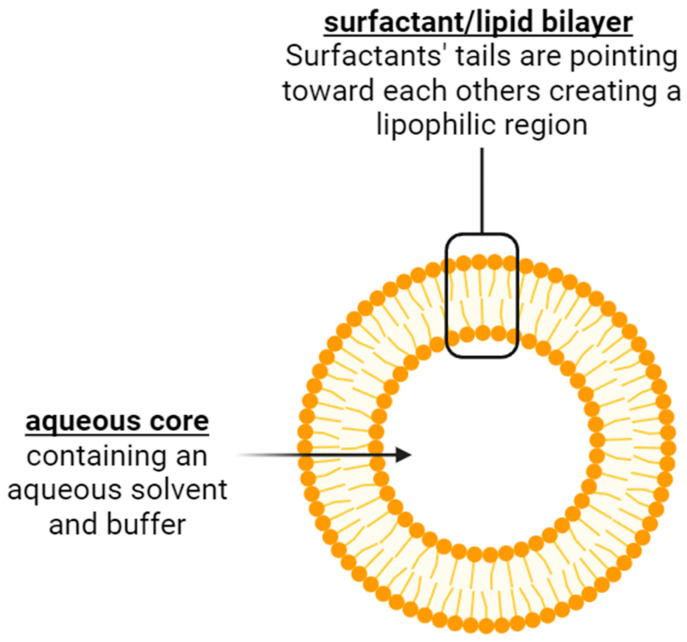
Structure of niosomes (created using BioRender).

Niosomes are nanovesicles consisting of a bilayer membrane and an aqueous core (Figure 1). The bilayer membrane is formed from non-ionic surfactant(s) and cholesterol lipid [1]. Co-surfactants such as Cremophor RH40 and Solutol HS-15 are sometimes added to stabilise the niosomal formulation and improve its properties [2,3].

Niosomes’ ability to entrap both hydrophilic and hydrophobic drugs, and the ability to formulate them using affordable materials and easy methods, has brought attention to their use [4]. Niosomes can be optimised to become a multi-route drug delivery system, where they can be administered orally [5], parenterally [6], topically [7], ocularly [8] and via other routes. They can also be optimised to carry drugs to specific targets (targeted drug delivery) and sustain the release of the active ingredient (modified drug release) [6]. The use of niosomes can reduce the side effects of certain medicines, such as chemotherapy, and protect the loaded drug from degradation by the body’s enzymes [4].

**Figure 2:**
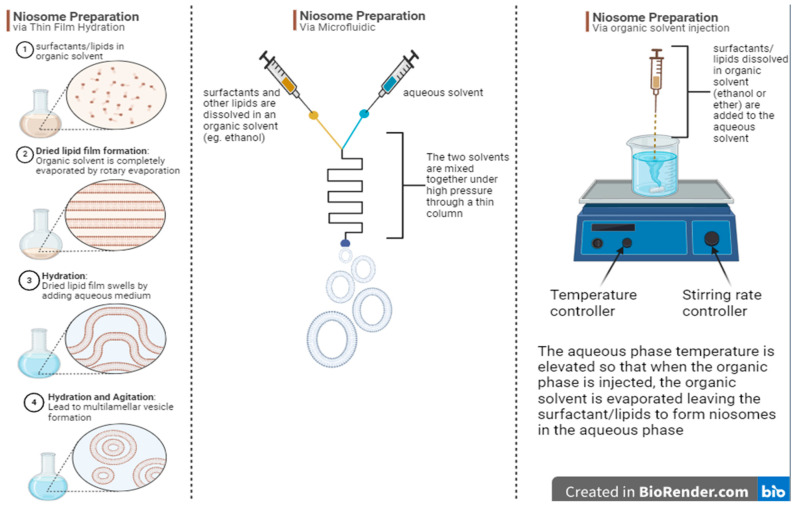
Three methods for formulating niosomes (thin-film hydration on the left, microfluidic in the middle and organic solvent evaporation techniques on the right) [9]. Created using BioRender.

There are several techniques used to formulate niosomes, such as thin-film hydration, microfluidic and organic solvent evaporation techniques [9]. A description of these methods is illustrated in Figure 2 below.

There are two methods of loading drugs into nanovesicles, which are passive and active drug loading. Passive loading relies on the niosomes to engulf the drug as they are forming, while active loading, which is the focus of this research, relies on changes in the niosomal suspension, which will drive the drug to reside inside the niosomes [10].

In the thin-film hydration technique, passive loading occurs when the loaded drug is added to the hydration media or mixed with the lipid mixture in the thin film and is randomly incorporated into niosomes during the hydration process. The forming niosomes will engulf the drug and encapsulate it within their aqueous core and/or between their bilayer membrane, depending on the drug’s hydrophilicity; hydrophilic drugs reside mainly in the aqueous core of niosomes, while hydrophobic drugs reside primarily in the bilayer membrane [1,10].

Depending on the drug being loaded, there are two main possible limitations to passive loading using the thin film hydration technique, which are percentage entrapment efficiency (%EE) [11] and drug stability under harsh conditions.

**Figure 3:**
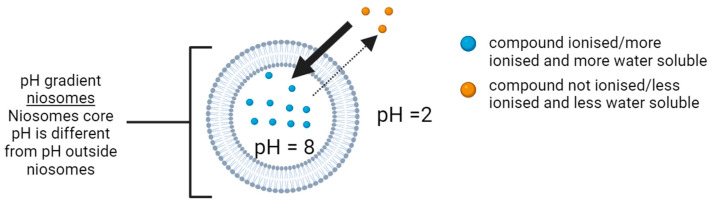
Enhancing entrapment efficiency via pH gradient. The pH inside the niosomes is different from that outside them. The drug will not be ionised outside the niosome and will only be ionised inside the niosomes. Ionised forms of the drug will prevent the drug from crossing the hydrophobic bilayer membrane, resulting in the accumulation of the drug in the niosomes. The net flow of the drug will be to the niosomes’ aqueous core. The figure was created using BioRender.

The active loading of drugs can solve these two issues. One of the main active loading methods is the pH gradient. In pH gradient niosomes (Figure 3), the pH outside the niosomes will be different from the pH inside the niosomes. This technique will help actively load compounds that have ionisable functional groups. The pH outside the niosomes will be adjusted such that it does not ionise the drug, while the pH inside the niosomes will ionise the loaded drug. The drug will cross the niosomes membrane in its less ionised form. Once it is inside the niosome, it will be entrapped. It will not leave the niosome because it is more ionised and more water soluble, which are two features that inhibit the drug from passing back through the hydrophobic niosome membrane. Thus, more of the drug will enter the niosomes, enhancing entrapment efficiency [4,9]. This technique is also used in liposomes [12].

To study the ability of niosomes to form pH gradients and enhance the %EE of ionisable compounds, a pH indicator will be used. The pH indicator will assess the ability of the niosomal membrane to maintain two separate environments with different pH levels (i.e., the niosome’s aqueous core and the external medium outside the niosomes). pH indicators are compounds that change their colour when exposed to different pH environments. They carry ionisable functional groups with known pKa values, and the colour change is linked to the ionisation state of the compound [13].

This research hypothesises that pH indicators can be used to study and visualise pH gradient niosomes, and they will have several advantages. For example, they can immediately indicate whether a pH gradient has been established across the niosomal membrane by observing the colour change in the suspension. They can be used as model drug molecules to represent different groups of active ingredients, and they can be easily detected in a spectrophotometer due to their unique colours and thus specific lambda max (λmax) that differentiate them from other compounds. Moreover, the change in colour of the solution can indicate the %EE. They can also be used to study the stability of the pH gradient by observing the change in colour over time. They can help in analysing the properties of niosomes and their membranes’ permeability.

**Figure 4:**
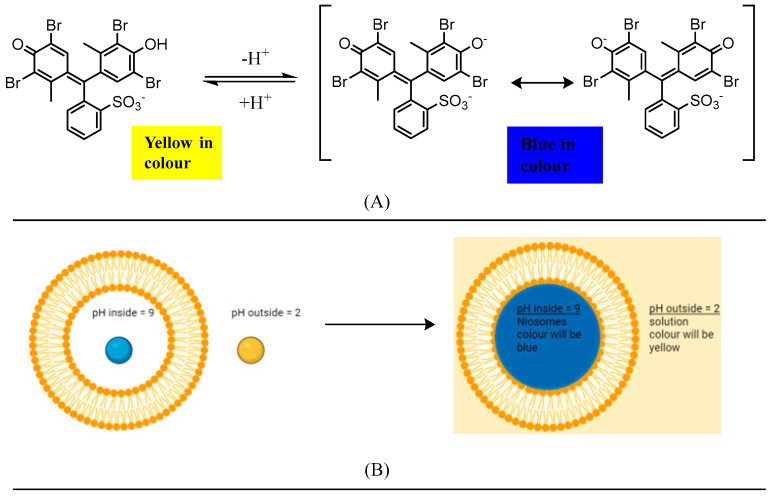
The effect of BCG ionisation on its colour and the colour of its surroundings. (**A**) Showing ionisation of BCG in different pH media (generated using ChemDraw 22.2.0). (**B**) The sphere represents a BCG compound where it is yellow in pH = 2 (pH < 3.8) and blue in pH = 9 (pH > 5.4). The environment that BCG is present in will change colour depending on its pH when pH gradient is established (created using BioRender).

In this research, the bromocresol green (BCG) pH indicator has been used to study the properties of pH gradient niosomes. BCG has a molar mass of 698.01 g/mol [14], it is sparingly water soluble and becomes di-anionic and blue at pH > 5.4, while mono-anionic is yellow at pH < 3.8, as shown in Figure 4A below [15].

When the pH inside the niosome is >5.4, BCG will become blue, and when the pH outside the niosome is <3.8, BCG will become yellow (Figure 4B). For that reason, there will be two colours present at the same time when pH gradient niosomes are obtained in the presence of BCG, where inside the niosome (pH > 5.4), it will become blue, while outside the niosome (pH < 3.8), it will become yellow (Figure 4B). As a result, the colour of the suspension will be green because mixing yellow and blue will give a green colour. The presence of green is an indication of the formation of pH gradient niosomes.

As more of the BCG enters the niosomes, the suspension colour will turn more blue than yellow. The shift in the colour can be an indication of the %EE of BCG and the success of the loading method. Measuring %EE can also be performed using the spectrophotometric method.

In this research, the most optimised niosomal formulation that maintains a pH gradient and entraps pH indicator BCG will be chosen to entrap doxorubicin via pH gradient loading.

**Figure 5:**
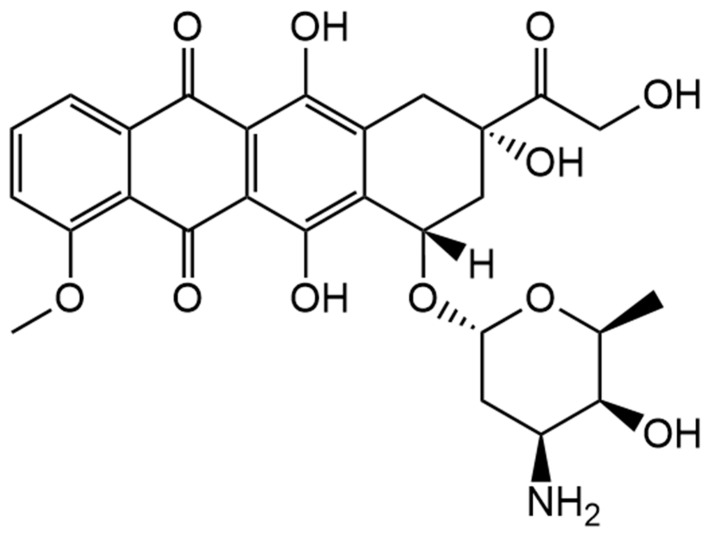
Structure of doxorubicin. Generated using ChemDraw 22.2.0.

Unlike BCG, doxorubicin is a basic drug. It has an amine functional group as shown in Figure 5 below. To load doxorubicin into niosomes via the pH gradient, the inside of the niosome must be acidic to allow the ionisation of the amine functional group when it reaches the core of the niosomes, while keeping the outside of the niosomes basic to de-ionise the amine. This way, doxorubicin will cross the niosome bilayer in its unionised form and become ionised when it reaches the acidic core and becomes entrapped [16]. Doxorubicin is a cytotoxic drug used in the treatment of different types of cancers such as breast cancer, lymphoma, leukaemia and others. It was found that encapsulating this drug into liposomes reduces its side effects, such as cardiotoxicity [17]. Thus, integrating it into niosomes can also minimise side effects [4]. 

Niosomes have several advantages over liposomes, including lower production costs and greater stability. In addition, lipids used in liposome production have the problem of variable purities, which is avoided in niosomes. These points make niosomes more favourable than liposomes [1]. Moreover, niosomes carrying doxorubicin can be designed to incorporate targeting moieties, allowing for the precise targeting of cancer cells and thereby enhancing the therapeutic efficacy of these nanovesicles [18,19]. Niosomes can carry multiple anticancer drugs to improve activity against multidrug-resistant cancer cells [1]. They can be designed to respond to various stimuli (such as pH, temperature, ultrasound, electric field, light and enzymes) that enhance the release of the drug at cancer sites [1].

While liposomes are well-studied, the potential of pH gradient-driven loading in niosomes remains underexplored. Two main knowledge gaps have been identified in the study of niosomes’ ability to actively load drugs using a pH gradient. The first gap is related to the lack of an analysis method to study and visualise pH gradients and drug loading in niosomes. For this purpose, the pH indicator bromocresol green (BCG) has been utilised in this research to load BCG into niosomes actively. The second gap is that no niosomal formulations have reached the market for medicinal product delivery. This research aims to formulate a novel niosomal formulation containing doxorubicin that enhances the accumulation of doxorubicin in cancer cells by specifically incorporating the co-surfactant Solutol HS-15, as will be explained later.

This research has two main objectives: the first objective is to study the ability of different niosomal formulations to actively load doxorubicin using a pH gradient. Because stable niosomes cannot be formed without the help of co-surfactants, Solutol HS-15, Cremophor RH 40 and Cremophor ELP were chosen for this research. The rationale for choosing these cosurfactants is as follows: Solutol HS-15 is known to inhibit the P-gp protein in cancer cells, thereby increasing the accumulation and toxicity of anticancer drugs. It also exhibits low haemolytic properties, indicating that it has minimal irritation and toxicity to healthy cells [20,21]. Also, Solutol HS-15 has a hydrophilic head consisting of 15 polyethylene glycol (PEG) molecules that can protect the niosomes from aggregation and stabilise the niosomes’ membrane. Cremophor RH 40 contains 40 PEG molecules in the head group, and Cremophor ELP has 35 PEG molecules [22]; hence, they were chosen to study the effect of large PEG groups on stabilising the pH gradient in niosomes. All three cosurfactants have not been used before in pH gradient niosomes. The second objective is to investigate how different buffers (Trizma, HEPES, hydrochloric acid and citric acid) and different loading temperatures can affect the stability of the pH gradient and active drug loading.

## 2. Materials and Methods

### 2.1. Materials

Doxorubicin HCl (Molecular weight: 579.98 g/mol), citric acid (Molecular weight: 192.12 g/mol) and HEPES (Molecular weight: 238.30 g/mol) were purchased from Flourochem (Hadfield, UK). Span 40 (Molecular weight: 402.57 g/mol) and Span 60 (Molecular weight: 430.62 g/mol) were obtained as free samples from Croda (Barcelona, Spain). Cremophor RH-40 (Molecular weight: ~2700 g/mol), Cremophor ELP (Molecular weight: ~2500 g/mol) and Solutol HS-15 (Molecular weight: ~960 g/mol) were obtained as free samples from BASF (Ludwigshafen, Germany). Bromocresol green (Molecular weight: 698.05 g/mol) was purchased from Sigma-Aldrich (Buchs, Switzerland), while cholesterol (Molecular weight: 386.65 g/mol) was purchased from Sigma-Aldrich (St Louis, MO, USA). Trizma base (Molecular weight: 121.14 g/mol), Trizma hydrochloride (Molecular weight: 157.60 g/mol), Chloroform (Molecular weight: 119.38 g/mol), Isopropanol (Molecular weight: 60.10 g/mol) and Hydrochloric acid (HCl) were purchased from Fisher Scientific (Loughborough, UK). Sephadex G50 was purchased from GE Healthcare (Uppsala, Sweden). All reagents were of analytical grade.

### 2.2. Niosomes Preparation

Niosomes were prepared using the thin film hydration technique. Surfactants (Span 60 or Span 40) and cholesterol with or without cosurfactants (Cremophor RH40, Cremophor ELP or Solutol HS-15) were used to formulate the niosomes. The ingredients (as summarised in Table 1 and Table 2) were dissolved in 3 mL of chloroform in a 25 mL round-bottom flask. The chloroform was then evaporated using a rotary evaporator (Rotavapor R-210, Buchi, Flawil, Switzerland) at 40 °C. The pressure was reduced to 400 mbar and kept for 5 min to warm the chloroform. Then, the pressure was gradually reduced to 200 mbar to ensure the evaporation of chloroform without the solvent bubbling. Once a thin film is formed, the round bottom flask containing the formed thin film is detached from the rotary evaporator and left in open air inside a fume cupboard to allow the thin film to dry overnight.

The thin films of BCG formulations (Table 1) were hydrated with a buffer of choice (Trizma or HEPES) at 60 °C for 1 h using a shaking water bath (NB-303 model, N-Biotek, Bucheon-si, South Korea). The thin films in formulations F1 to F5 were hydrated with 5 mL of 0.1 M Trizma buffer (pH 9.0) containing BCG at a concentration of 0.1 mg/mL to assess the efficiency of passive drug loading. However, in formulations F6 to F13, the formulations were hydrated with 4.5 mL of Trizma or HEPES buffer without adding BCG to test for active loading of BCG by adding the drug after the formation of niosomes. All BCG formulations were prepared in triplicate.

Unless otherwise stated, the suspension was then sonicated using an ultrasound bath sonicator (Hilsonic, Birkenhead, UK) for 30 min to change the niosomes’ shape and size from multilamellar vesicles (MLV) to large unilamellar vesicles (LUV).

Doxorubicin formulations (Table 2) were treated slightly differently. The F1-D thin film was hydrated with 5 mL of Trizma buffer to test for the passive loading of doxorubicin. However, formulations F2-D to F5-D and F5-D-E were hydrated with an ammonium sulfate solution (0.12 M or 0.25 M). All doxorubicin formulations were subjected to bath sonication after the thin film hydration and before the addition of doxorubicin using an ultrasound bath sonicator (Hilsonic, Birkenhead UK) for 30 min to convert the multilamellar vesicles (MLVs) to large unilamellar vesicles (LUVs). To study the effect of extrusion on the niosomal size and size distribution, formulation F5-D-E was extruded using a mini-extruder (Avanti, Alabaster, AL, USA) with a 100 nm filter. The formulation was heated to 40 °C and passed through the filter 11 times. All formulations were prepared in duplicate, as consistent results were obtained.

### 2.3. Drug Loading

Drug loading techniques were different between BCG and doxorubicin.

#### 2.3.1. Bromocresol Green, BCG, Loading

A 100 mg% BCG solution was prepared in 0.1 M Trizma buffer (pH 9.0). The BCG drug model was either loaded into niosomes by adding 0.5 mL of BCG solution to 4.5 mL of hydration media (for passive loading of niosomes like in formulations F1 to F5) or by adding the BCG solution to the niosomal suspension after niosomes were formed (for active loading of niosomes like in formulations F6 to F13 by adding the drug after the formation of niosomes).

Achieving pH gradient and thus starting active loading of BCG into niosomes was performed by the addition of either 0.1 mL of 1 M HCl to 1 mL of the formulation containing BCG (like in formulations F1–F7, F10 and F12–F13) to reduce the pH to 1.5 (for formulations hydrated with Trizma buffer) and to 2.8 (in the case of formulation F10, which is hydrated with HEPES buffer) or adding 0.5 mL of 0.25 M citric acid to 1 mL of the formulation containing BCG (like in formulations F4, F5, F8, F9 and F11) to reduce pH to 3.2 (for formulations hydrated with Trizma buffer) and to 3.36 (in the case of formulation F11, which is hydrated with HEPES buffer). Drug loading took place at either room temperature (RT) of around 23 °C or at 40 °C.

In formulations F1 to F3, active loading was also tested by adding 0.1 mL of 1 M HCl to 1 mL of the formulation, which already contained BCG (0.1 mg/mL) and was passively loaded. HCl was added either before the formulations were sonicated to test the efficiency of active loading via pH gradient on large MLV niosomes or after the formulations were sonicated to study the effect of reducing niosomes’ size on %EE via active loading.

For formulations F4 and F5, either 0.1 mL of 1 M HCl was added to 1 mL of the formulation to reduce the pH to 1.5 or 0.5 mL of 0.25 M citric acid was added to 1 mL of the formulation to reduce the pH to 3.2, creating a pH gradient.

#### 2.3.2. Doxorubicin Loading

For passive loading in formulation F1-D, 1 mL of the formulation was taken and diluted to 5 mL to maintain a similar concentration of the niosomes’ ingredients to that in the pH gradient niosomes. Then, 0.9 mL of the formulation was taken and mixed with 0.1 mL of a doxorubicin solution (1 mg/mL dissolved in 0.15 M NaCl solution). Drug loading was left for 1 h at room temperature, RT.

To create a pH gradient, the Sephadex G50 gel column technique was used. The Sephadex G50 column was prepared by mixing 1 g of Sephadex G50 powder with 20 mL of either PBS buffer (pH 7.4, the buffer formed from sodium chloride 0.137 M, potassium chloride 0.0027 M, sodium phosphate dibasic 0.01 M and potassium phosphate monobasic 0.0018 M) or Trizma (0.1 M, pH 9.0). The suspension was then left to equilibrate overnight at room temperature, allowing the Sephadex resin to swell. The suspension was then loaded into a column. The pH gradient was created by passing 1 mL of the niosomal formulation through the Sephadex column pre-equilibrated with either PBS (as in formulation F2-D) or Trizma (as in formulations F3-D, F4-D, F5-D and F5-D-E). Then, 5 mL of the new pH gradient niosomal formulation was collected.

To load doxorubicin, 0.9 mL of the pH gradient formulation was taken and mixed with 0.1 mL of doxorubicin solution (1 mg/mL dissolved in 0.15 M NaCl solution). Drug loading was left for 30 or 60 min at RT or 60 °C.

### 2.4. Bromocresol Green Lambda Max and Calibration Graph

Lambda max was measured using a UV/Vis spectrophotometer (Spectrasonic Camspec Ltd., Leeds, UK). A wavelength scan was performed on a BCG solution in Trizma buffer (0.1 M, pH 9.0) (blue colour) to identify the λmax between 190 nm and 900 nm.

**Figure 6:**
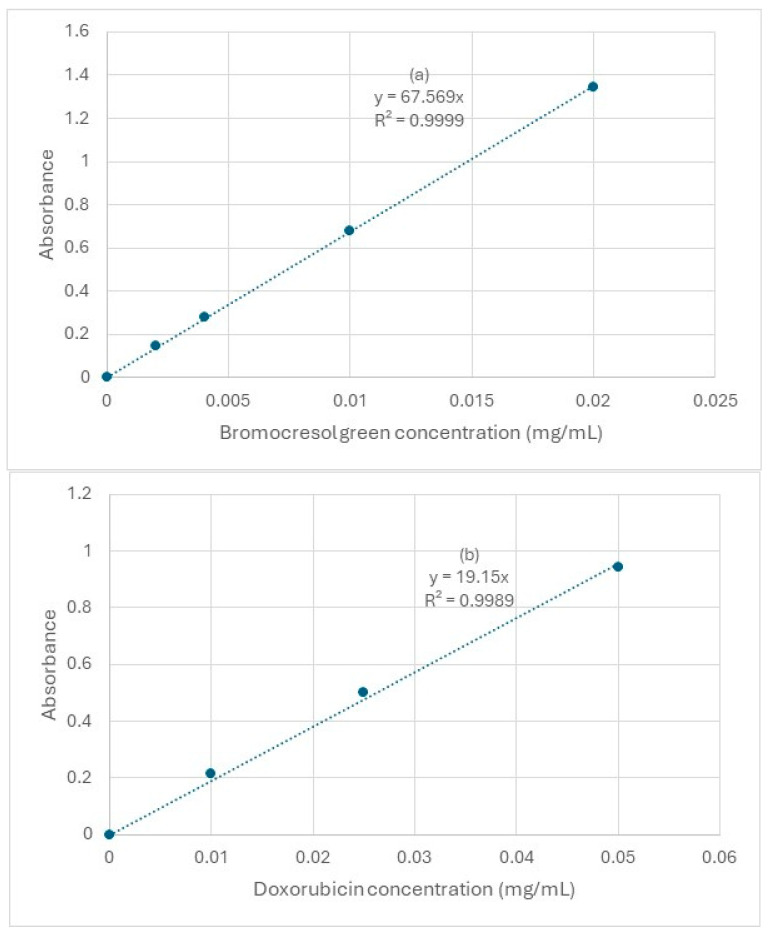
(**a**) Calibration graph for BCG in its blue form measured at 617 nm wavelength. (**b**) Calibration graph for doxorubicin measured at 482 nm wavelength (n = 3).

A calibration graph was then obtained by measuring the absorbance of different concentrations of BCG solution. The absorbance at λmax (617 nm) of four BCG standard solutions was measured (Figure 6a). All standards were prepared in a 1:1 ratio of Trizma buffer (0.1 M, pH 9.0) to isopropanol to mimic the conditions of the samples when measuring BCG entrapment efficiency.

### 2.5. Doxorubicin Lambda Max and Calibration Graph

The doxorubicin lambda max was determined at pH levels of 9.0 and 5.5. Doxorubicin was dissolved in Trizma buffer (0.1 M, pH 9.0) or 0.15 M NaCl solution (pH 5.5) to produce a concentration of 0.025 mg/mL. Knowing from the literature that doxorubicin lambda max is around 480 nm [23], a lambda max search was conducted between 190 nm and 650 nm.

The λmax was identified for doxorubicin in the two solutions to be 482 nm. Since isopropanol will be used to disrupt the niosomes’ bilayer and release the drug, doxorubicin was dissolved in 0.1 M Trizma with isopropanol (a 1:1 ratio) to obtain the calibration curve (Figure 6b).

### 2.6. Bromocresol Green Entrapment Efficiency

The method used to investigate the %EE was based on determining the total drug concentration in the formula, rather than relying solely on the theoretical drug quantity, for an accurate measurement of the %EE. Hence, for pH gradient niosomes, 0.5 mL of the suspension was taken at 15 and 60 min intervals during drug loading and mixed with 0.5 mL of the same buffer used to hydrate the niosomal thin film to quench the pH gradient loading by raising the pH to above 5.4. Then, the 1 mL suspension was transferred to a 2 mL Eppendorf tube and centrifuged using a Centrifuge Mikro 200R (Hettich Zentrifugen, Tuttlingen, Germany) at 15,000 RPM for 1 h at 4 °C to allow the niosomes to pellet and separate from the supernatant [24]. Then, 0.5 mL of the supernatant containing the unentrapped drug was taken and added to 1.5 mL of isopropanol. The absorbance of this new solution was measured, and the concentration of the free BCG was calculated. It was found that a 1:1 and 1:3 ratio of Trizma to isopropanol gives similar absorbance results. However, increasing the amount of isopropanol by using a 1:3 ratio of Trizma to isopropanol was found to be more effective at disrupting the niosomes when measuring the total BCG amount in the formulation.

To measure the total amount of BCG model drug in the formulation loaded via active loading, 0.5 mL of the niosomal suspension was taken and mixed with 0.5 mL of the same buffer used to hydrate the niosomal thin film. Then, 0.5 mL of the suspension was taken and added to 1.5 mL of isopropanol to disrupt the niosomes and release the entrapped drug. A homogeneous solution was obtained. The absorbance of the formed solution was measured, and its BCG concentration, the actual total concentration in the formula, was calculated. To avoid any possibility of BCG instability, the total concentration of the drug in the formulation was measured at the beginning of the experiment as BCG solutions and after drug loading as a noisome suspension to assess drug retention and encapsulation efficiency.

For passively loaded niosomes, 0.5 mL of the suspension was taken and mixed with 0.5 mL of the same buffer used to hydrate the niosomal thin film to dilute the sample for absorbance measurement purposes. The 1 mL suspension was added to 2 mL Eppendorf tubes and centrifuged under the same conditions as above for active loading.

To measure the total amount of drug in the niosomes’ formulation loaded passively, the same steps applied above for actively loaded niosomes were employed.

The percentage of entrapped BCG was measured using the following equation:

$$\%\mathrm{EE}=(1-\frac{concentration\;of\;drug\;in\;supernatant}{total\;concentration\;of\;drug})\times 100$$

All measurements were conducted in triplicate.

### 2.7. Measuring Doxorubicin Entrapment Efficiency

Entrapment efficiency was measured by comparing the concentration of the drug that was unentrapped against the total concentration of the drug in the formulation as a whole.

The total concentration of the drug in the formulation was measured at the beginning of the experiment as solutions and after drug loading to assess drug retention and loading efficiency and to detect/avoid any possibility for drug instability. For this, 0.1 mL of the formulation was taken and mixed with 1 mL of an isopropanol solution. After disrupting the niosomes with isopropanol, 0.9 mL of 0.1 M Trizma buffer was added. The absorbance was then read at λmax 482 nm to determine the actual total drug concentration in the formula.

To measure the concentration of the unentrapped drug, 0.15 mL of the formulation was taken at 30 and 60 min during drug loading. The collected niosomal suspension was pelleted via centrifugation for 1 h at 15,000 RPM and at 4 °C. Then, 0.1 mL of the supernatant was collected and mixed with 1 mL of isopropanol and 0.9 mL of 0.1 M Trizma buffer. The absorbance was taken at λmax of 482 nm.

The percentage of entrapped doxorubicin was measured using the %EE equation mentioned in Section 2.6. All measurements were conducted in duplicate.

### 2.8. Physical Size Using Zetasizer

The niosomal size was measured using Zetasizer ZSP (Malvern Instruments, Malvern, UK). Each sample was diluted 50 times with the same buffer that the sample contained. All experiments were conducted in triplicate.

### 2.9. Niosomes Morphology

Light microscope Bioblue.Lab (Euromex, Duiven, The Netherlands) was used to observe niosomes morphology under 100× magnification.

### 2.10. Statistical Analysis

Statistical significance was calculated using a *t*-test, where *p* < 0.05 indicates a significant difference.

## 3. Results and Discussion

### 3.1. Finding Lambda Max and Calibration Graph

A wavelength scan showed that when BCG is in its blue form (pH > 5.8), its λmax is 617 nm. It should be noted that the wavelength scan graph is entirely different when BCG is in its yellow form (pH < 3.8), where its lambda max becomes 444 nm. This is because when a chemical substance changes colour, it interacts with visible light differently. For this reason, when measuring entrapment efficiency and when establishing a calibration graph, the colour of BCG should be either wholly yellow or completely blue. In this study, BCG will always be in its blue form when measuring its concentration.

### 3.2. Passive Loading of BCG into Niosomes

BCG was passively loaded into three niosomal formulations (F1, F2 and F3) of Span 60 and cholesterol, containing Cremophor RH40, Solutol HS-15 or Cremophor ELP. The formulation content (F1 to F3) is summarised in Table 1.

The size and entrapment efficiency of the three formulations were studied both before and after bath sonication. The results are summarised in Table 3 below.

As shown in Table 3 above, the %EE is very low when passive loading is used. This indicates that the entrapment efficiency of negatively charged large hydrophilic compounds (BCG molar mass = 698.01 g/mol [14]) at a formulation concentration of 12 micromoles/mL, using Span 60 surfactant, can be very low.

This makes BCG a suitable candidate for studying the effect of a pH gradient (active loading) on enhancing the %EE of such compounds.

After sonicating the formulations containing BCG, leakage of BCG occurred in all formulations. This is one of the limitations of passive loading drugs, where formulation optimisation can result in a reduction in %EE. Using probe sonication is no different to bath sonication, where Yeo et al. showed that the entrapment efficiency of methylene blue, a hydrophilic drug, significantly decreased after the use of probe sonicator applied at an amplitude of 40% for two cycles of 2 min with 1 min rest in between (in one formulation, %EE was 40.1% before sonication and became 11.6% after sonication) [11].

One of the advantages of sonication is the reduction in niosomal size. As shown in Table 3 above, bath sonication has significantly reduced particle sizes and decreased the polydispersity index (PDI) considerably in all three formulations (*p* < 0.05).

### 3.3. Studying the Ability of Niosomes to Form a pH Gradient Using BCG and the Effect of the pH Gradient on the %EE of BCG

**Figure 7:**
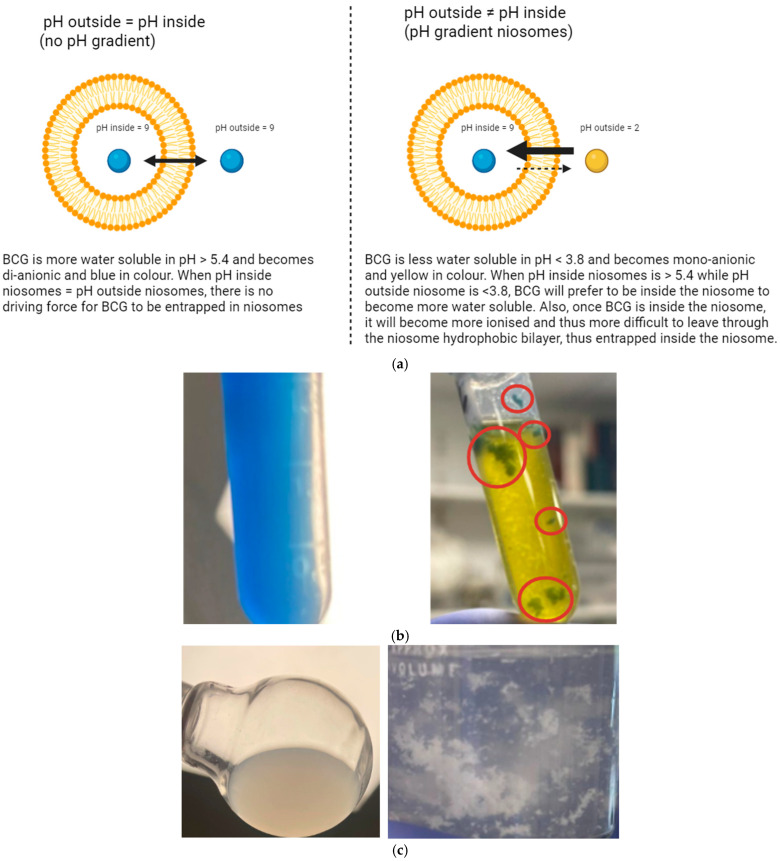
(**a**) pH gradient is the driving force for entrapping BCG. (**b**) F4 formulation, produced with Span 60 and cholesterol, without co-surfactant. F4 was prepared in Trizma buffer (0.1 M, pH 9) containing 0.1 mg/mL BCG (blue suspension—pH 9). The suspension turned yellow after the addition of citric acid (yellow—pH 3.2), where green aggregates (shown in red circles) can be seen clearly in the yellow suspension, meaning that niosomes are not stable without a co-surfactant. (**c**) On the left, Span 60-cholesterol niosomal suspension after sonication in Trizma buffer (0.1 M, pH 9.0) without BCG. On the right, the same niosomal suspension after addition of HCl to reduce pH to 4.0, only aggregates could be seen, indicating the niosomes are not stable in acidic conditions without co-surfactants, and the instability is not due to the presence of BCG. The same observation was seen with Span 40-cholesterol niosomes.

To date, there has been no publication that has actively loaded BCG to study the ability of niosomes to create and maintain a pH gradient. Active loading of BCG into niosomes can be achieved via pH gradient because BCG will become more water soluble and di-anionic in pH > 5.4, while less water soluble and mono-anionic in pH < 3.8 [15]. When the pH inside the niosome is greater than 5.4, BCG will enter the niosome and become trapped because it cannot cross back through the niosomal hydrophobic bilayer due to the di-anionic charge it acquires at pH levels above 5.4. This technique will force the BCG to enter and remain inside the niosomes. This technique is illustrated in Figure 7a.

The effect of different co-surfactants on the ability of niosomes to form a pH gradient was studied. Niosomes were formulated using Span 60 or Span 40, along with cholesterol, with or without a co-surfactant such as Cremophor RH40, Cremophor ELP or Solutol HS-15.

#### 3.3.1. Formulations of pH Gradient Niosomes Containing Span 60 or Span 40 with Cholesterol Only (F4 and F5)

It was not possible to form stable niosomes with formulations containing only Span 60 and cholesterol, where immediately after reducing the pH, the niosomes aggregated to create big pieces that are visible in the suspension (Figure 7b).

The same observation was noted when the Span 40-cholesterol formulation (F5) was used to determine if it was the Span 60 that caused the aggregation to occur. Also, the same problem occurred when using HCl instead of citric acid.

The aggregation observation was also tested in a less acidic pH, where the pH was reduced to 4.0 without the use of BCG, to ensure that it is not BCG that causes aggregation or the very low pH that is responsible for this phenomenon. The same aggregation observation was obtained (Figure 7c).

A similar observation was obtained in the work of Dehaghi et al., where they stated that reducing pH to 6 and 5.5 will prevent the formation of niosomes containing Span 60 and cholesterol [25]. They have also noted that the concentration of the buffer used can also affect the stability of the niosomes. They used di-ammonium hydrogen phosphate buffer to hydrate the surfactant-cholesterol thin film and found that when the buffer concentration was 200 mM, niosomes formed at a pH of 6.5. However, niosomes did not form when the buffer concentration was 250 mM, even though the pH was also 6.5.

#### 3.3.2. Effect of Co-Surfactant Used and Niosomal Size on Entrapment Efficiency Using pH Gradient

The advantages of using the pH gradient drug loading technique are that it increases the niosomes’ %EE [12,26] without exposing the drug to harsh conditions that can degrade it, such as high temperature during thin film hydration and sonication during size reduction. Also, increasing entrapment efficiency can reduce the amount of niosomes needed to achieve the same therapeutic dose. Moreover, the drug will be added after the formation and optimisation of the niosomes, which allows the optimisation of the niosomes first, so that the drug does not leak after entrapment (for example, the sonication of niosomes to optimise their size can result in drug leakage and reduction of %EE) [11].

In this study, to confirm that a pH gradient has occurred, two criteria must be met. First, the colour of the suspension should initially turn yellow after the pH is reduced to less than 3.8, but then turn green or blue. Also, the %EE should be enhanced.

The colour should turn green-blue because the pH inside the niosome aqueous core is >5.4, while the pH outside the niosome is <3.8, which causes BCG to become blue inside the niosomes while becoming yellow outside the niosomes. When blue and yellow colours are present in the same suspension, a green colour is obtained. If entrapment efficiency is very high, a blue colour should be obtained because most of the BCG is in niosomes at pH levels above 5.4.

**Figure 8:**
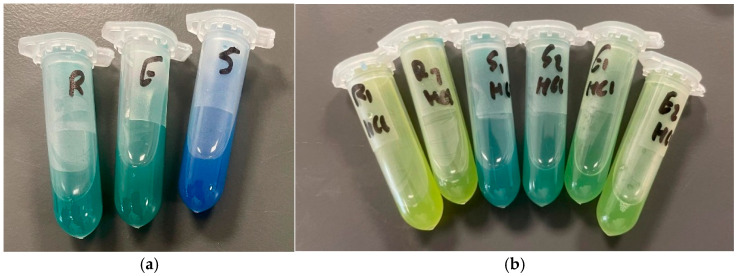
(**a**) The change that occurred in three formulations (from left to right: F1, F2 and F3) after 6 min of adding HCl. HCl was added to these formulations before they were sonicated. Note that immediately after adding HCl, the suspension turned yellow; then, it gradually changed in colour to green and blue (from left to right). The %EE reported after 15 min of drug loading is F1 = 55.73%, F2 = 41.45% and F3 = 57.45%. (**b**) The change that occurred in three sonicated formulations (from left to right: two repeats for F1, two repeats for F3 and two repeats for F2) upon the addition of HCl. HCl was added to these formulations after they were sonicated. The picture was taken 15 min after adding 0.1 mL of 1 M HCl. %EE reported after sonication after 15 min of drug loading is F1 = 15.57%, F2 = 17.81% and F3 = 67.86%.

Co-surfactants (Cremophor RH40, Cremophor ELP and Solutol HS-15) enhanced the stability and prevented the aggregation of niosomes upon pH reduction. All three cosurfactants contributed to the formation of niosomes that can establish a pH gradient across their membranes. However, the effect of the different co-surfactants on entrapment efficiency and pH gradient was different. Figure 8a below shows the colour changes after the addition of HCl to formulations F1, F2 and F3 before they were sonicated.

As mentioned in the introduction, two advantages of using BCG to study pH gradients are that it immediately indicates whether a pH gradient has been established across the niosomal membrane, and the change in colour of the solution can provide an indication of the %EE.

After the addition of 0.1 mL of 1 M HCl, the colour of the suspension should become completely yellow because the pH will be 1.5. As shown in Figure 8a, a pH gradient has developed in all three formulations, as F1 and F2 turned green while F3 turned blue after 6 min of HCl addition. It can also be predicted that entrapment efficiency will be highest in F3 because it produced the bluest colour. %EE results are summarised in Table 4 below.

Formulation F3 showed the bluest colour in Figure 8a, but it yielded an entrapment efficiency close to that of F1 (Table 4, 57.45% vs. 55.73%, respectively, *p* = 0.277). This may be because upon pelleting the niosomes via centrifugation, some of the drug leaked out of the niosomes. Leakage can happen because niosomes will be exposed to high forces during centrifugation, and pelleting can cause niosomes to merge, resulting in leakage [27,28]. More leakage occurred in F3 than in the other formulations due to the theoretically high concentration of BCG inside F3 niosomes.

The effect of niosomal size on %EE has also been studied. After reducing the niosome size using bath sonication to convert the niosomes from MLV to LUV, the %EE using a pH gradient was also measured in the smaller niosomes. The colour change of the formulation after the addition of HCl to the three sonicated formulations was recorded in Figure 8b. Niosomal size and entrapment efficiency results are summarised in Table 4 below.

As can be seen in Figure 8b, the colours of formulations F1 and F2 after sonication and after 15 min of active drug loading are more yellow than blue, which can indicate that these formulations may lose their pH gradient quickly and are less able to entrap BCG via pH gradient once their size is reduced.

The results in Table 4 for the pH gradient after sonication match the colour change obtained in Figure 8b above. Among the three formulations, F3 showed the bluest colour after 15 min, and it provided the highest %EE (*p* < 0.05). This further supports the advantage of using BCG to study pH gradient, where colour change can be an indication of %EE.

**Figure 9:**
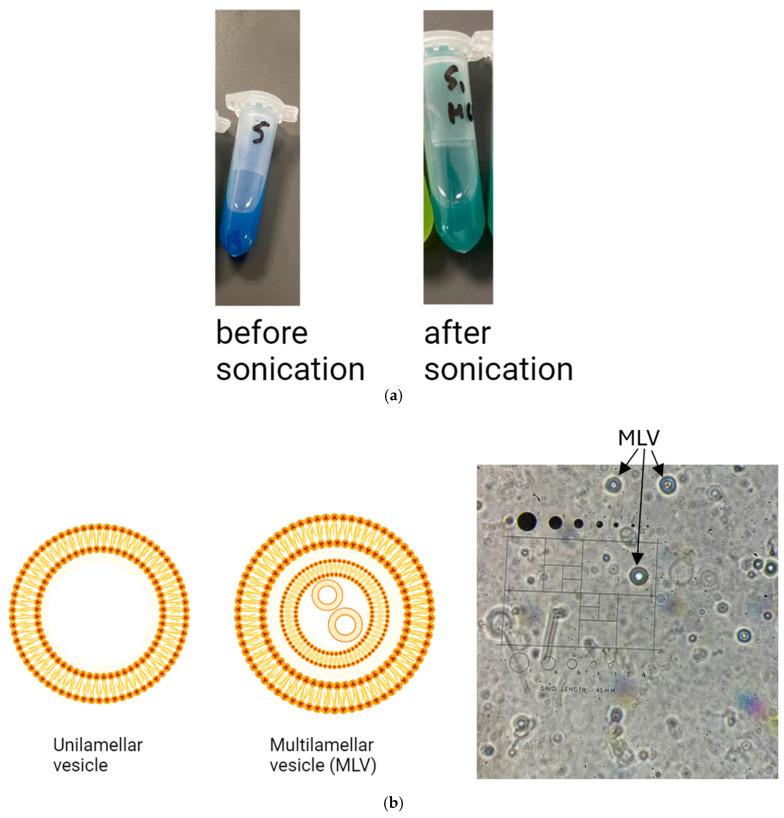
(**a**) Comparison of F3 colour after addition of HCl to the formulation before sonication (left picture) (%EE after 15 min of drug loading is 57.45%) and after sonication (right picture) (%EE after 15 min of drug loading is 67.86%). The formulation is bluer before sonication but shows lower entrapment efficiency. (**b**) Structure of unilamellar vesicles and multilamellar vesicles (MLVs), F3 before sonication, under the light microscope (100× magnification).

It was noted that after the addition of HCl, niosomes with a larger size (Figure 8a) were much bluer than niosomes with a smaller size (Figure 8b), yet maximum entrapment efficiency in F3 before sonication is less than that after sonication (57.45% vs. 67.86%, respectively, *p* < 0.05). This can be explained by the method used to separate entrapped BCG from unentrapped BCG, which is pelleting via centrifugation. As discussed previously, during centrifugation, some niosomes may be deformed due to the high gravitational force to which they are exposed, resulting in leakage of their BCG. Large niosomes are at more risk of being damaged than smaller niosomes (the larger surface area of individual large niosomes makes them more exposed to friction and collision with surrounding objects). This could have resulted in the %EE being recorded as higher in the F3 formulation after sonication than before sonication, even when the colour is bluer in the F3 formulation before sonication than after sonication, as shown in Figure 9a below, which compares the two sizes head-to-head.

It can be inferred from Figure 9a, based on the intensity of the blue colour for F3 before and after sonication, that large MLV niosomes produced a higher %EE. The reason behind this larger %EE is related to the surface-area-to-volume ratio. Another reason is related to the structure of niosomes before and after sonication. Similar to liposomes, niosomes can lose their pH gradient, and if this happens during the active drug loading phase, the %EE will be reduced [29]. Larger niosomes have a smaller surface-area-to-volume ratio than smaller niosomes and also have a larger aqueous core volume, which makes them more resistant to losing their core pH and maintaining a pH gradient for a longer time. The added HCl will find it more challenging to enter the niosomes when the surface area available is smaller. Thus, larger niosomes can maintain a pH gradient more effectively than smaller niosomes and can therefore achieve a higher %EE.

Another explanation is related to the structure of niosomes before sonication. The thin-film hydration method for forming niosomes is known to form multilamellar vesicles (MLVs) [30]. MLVs are niosomes that form vesicles inside vesicles (Figure 9b). It will be more challenging for hydrogen ions from HCl to cross the MLV membrane layers and change the pH of the multiple cores of the MLVs. The different layers of MLVs will slow the diffusion of H^+^ ions, thereby maintaining the pH gradient for a longer period, which enables higher entrapment efficiency. Therefore, there will be multiple environments that BCG can partition into, where it is shielded from acidic pH, minimising its exposure to protonated conditions. MLVs were observed under a light microscope, as shown in Figure 9b.

It should also be noted that in our preliminary data, sonicating actively loaded MLVs resulted in the loss of BCG. That is why, in this research, MLVs are sonicated first to produce LUVs, and then BCG is actively loaded into the smaller niosomes, rather than sonicating the actively loaded MLVs, as the %EE will be very low.

#### 3.3.3. Rationalising the Effect of Co-Surfactants on Niosomal Stability During pH Gradient Loading

**Figure 10:**
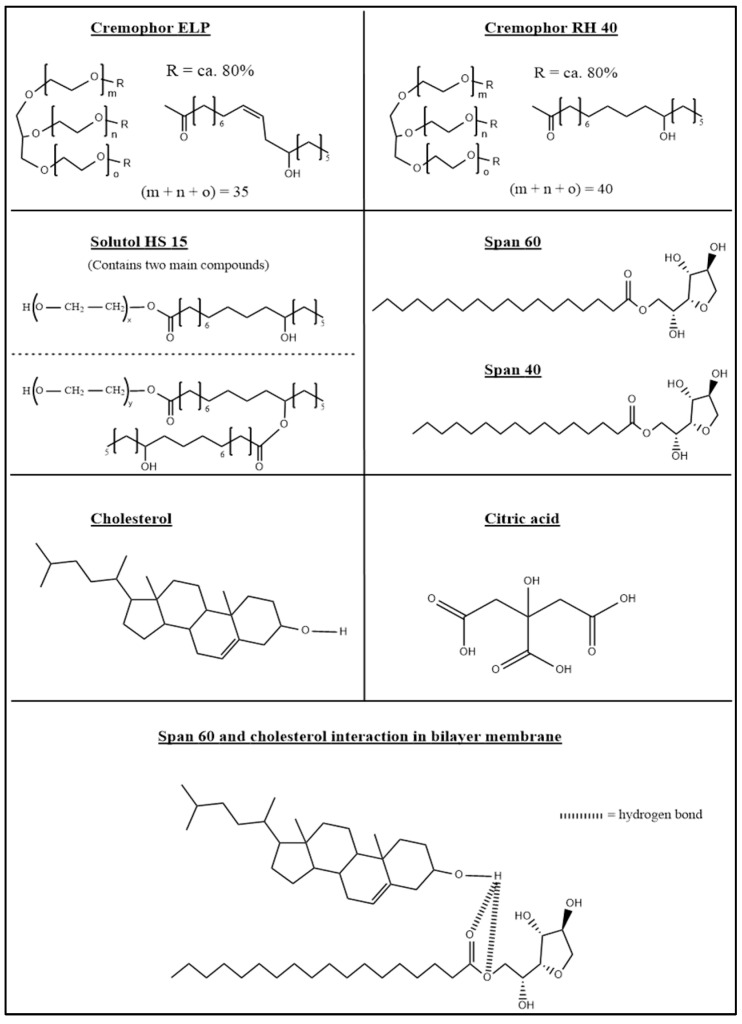
Structure of co-surfactants (Cremophor ELP, Cremophor RH40 and Solutol HS-15), main surfactants (Span 60, Span 40), cholesterol and citric acid. The figure also illustrates how Span 60 and cholesterol interact in niosomes [32]. Note that one hydrogen atom cannot form hydrogen bonds with two oxygen atoms at the same time. However, this figure illustrates that the oxygen atoms with which the hydrogen atom can form hydrogen bonds are one at a time. All structures were generated using ChemDraw 22.2.0.

As previously discussed, niosomes formed using only Span 60 or Span 40 and cholesterol are not stable in acidic media. Niosomes’ surface is rich in hydroxyl groups (OH) because each Span surfactant molecule has three hydroxyl groups in its hydrophilic head (Figure 10). This means the surface is negatively charged due to its high content of electronegative oxygen atoms. Niosomes tend to aggregate because they might collide with each other as they move in the suspension. The negativity of the niosomes’ surface will prevent aggregation and fusion, as the repelling effect of negative charges on the surface will cause niosomes to repel each other.

Reducing the suspension pH value will increase the number of H^+^ ions in the suspension, which can theoretically reduce the negativity of the niosomes’ surface and could potentially bring it close to neutrality. Thus, when niosomes collide, their surfaces will not be negatively charged and will not repel each other, resulting in the niosomes merging. What further supports this theory is our finding that niosomes disaggregate when the suspension pH is increased and the fact that the aggregations are irreversible when the aggregates are left for one hour in acidic media.

Adding co-surfactants will stabilise the niosomes in acidic media because they will prevent their merging by forming a physical barrier at the surface of the niosomes. The co-surfactants shown in Figure 10 below will use their hydrophobic tail to anchor themselves in the niosomes next to the Span surfactants. The length of the hydrophobic tail in the Cremophor RH 40, Cremophor ELP and Solutol HS-15 co-surfactants is 17 carbon atoms, which is similar to that of Span 60. The hydrophilic heads will extend in front of the niosomes’ surface to form a hydrophilic chain. The size of the hydrophilic head depends on the cosurfactant used, as shown in Figure 10 below. Cremophor RH 40 contains 40 polyethylene glycol molecules in the head group, Cremophor ELP contains 35 polyethylene glycol groups and Solutol HS-15 contains 15 polyethylene glycol groups [22].

The results in Table 4 above show that Solutol HS-15 with Span 60 and cholesterol has a better ability to entrap the drug in niosomes via the pH gradient method than Cremophor RH40 and Cremophor ELP. This can be attributed to the relatively smaller size of Solutol HS-15 and its more compatible structure with Span 60 surfactant, as shown in Figure 10. Solutol HS-15 has one hydrophobic tail, similar to Span 60, which has 17 carbon atoms. This can allow Solutol HS-15 to blend well with the Span 60 surfactants in the niosomal bilayer membrane.

Cremophor RH40 and Cremophor ELP have three hydrophobic tails, which can disturb the niosomal membrane because they might not be compatible with Span surfactants, as Span 60 has only one hydrophobic tail. In addition, Cremophor RH40 and Cremophor ELP sizes and molar masses (~2700 g/mol and ~2500 g/mol, respectively) [22] are much larger than Span 60 (430.62 g/mol) [31], which can, in theory, make them less compatible with Span 60 and thus form less stable niosomal membranes.

#### 3.3.4. Co-Surfactants Cannot Maintain a pH Gradient Without Niosomes

**Figure 11:**
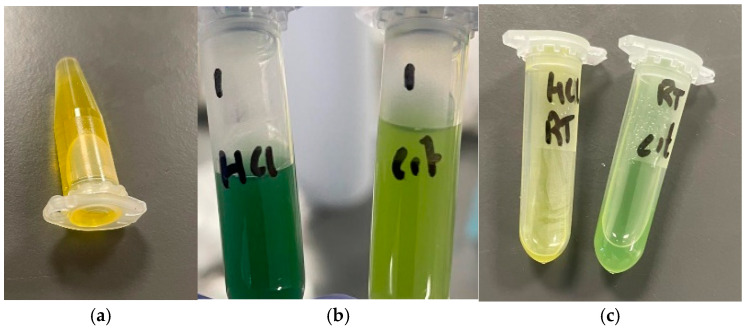
(**a**) Span 60-cholesterol-Solutol HS-15 added directly to water and sonicated with BCG 0.1 mg/mL after the addition of HCl (0.1 mL of 1 M). The suspension turned and remained yellow. (**b**) The F6 formulation after 15 min of adding HCl (left Eppendorf) and the F8 formulation after 15 min of adding citric acid (right Eppendorf). %EE after 15 min for F6 is 68.59% and for F8 is 14.86%. (**c**) The F6 (left Eppendorf) and F8 (right Eppendorf) formulations after 60 min of adding HCl (left Eppendorf) and citric acid (right Eppendorf). %EE after 60 min drug loading under pH gradient for F6 is 34.61% and for F8 is 29.63%.

To demonstrate that the formation of niosomes enables the creation of pH gradients, rather than, for example, micelle formation by cosurfactants, F3 was prepared by adding the ingredients directly to water (without thin-film hydration) and sonicating them using a bath sonicator. Upon the addition of HCl solution, the suspension turned yellow immediately and did not produce a green colour (Figure 11a).

#### 3.3.5. Other Uses of BCG in Studying Niosomes

It can be concluded from Section 3.2, Section 3.3.1, Section 3.3.2, Section 3.3.3 and Section 3.3.4 that BCG can be used to study the properties of niosomal membranes and the ability of different surfactants and cosurfactants to form stable niosomes that can carry drugs and keep them separate from the external environment. It can also study the permeability of different niosomal membranes made from various ingredients, as shown below.

#### 3.3.6. Addition of BCG and Actively Loading It into Niosomes After Optimising Niosomes

In many cases, to achieve high entrapment efficiency using a passive loading technique, a high concentration of niosome ingredients may be required, which can increase the risk of side effects from the formulation when administered to patients. For example, when pegylated liposomes are administered, they can cause a dose-limiting side effect called palmar-plantar erythrodysesthesia, resulting from the accumulation of liposomes in the skin capillaries [17]. Thus, unnecessarily increasing the number of nanovesicles can increase their side effects. In addition, niosomes formed using the thin film hydration technique are typically very large in size [3,11] and cannot be administered parenterally. Reducing the size of the niosomes, for example, using the sonication technique, can result in significant leakage and thus a significant reduction in entrapment efficiency [11]. Low entrapment efficiency can be problematic, resulting in the formulation’s inability to achieve therapeutic doses.

Another problem with passive loading is the exposure of the drug to extreme conditions that can result in the degradation of sensitive drugs. For example, the hydration of a thin film containing Span 60 requires a temperature above the Span 60 gel-fluid transition temperature [1], which is 53 °C [33]. Some drugs are heat-sensitive, and if left in water at a high temperature, they can be degraded, such as drugs containing ester functional groups, which can undergo ester hydrolysis [34].

To overcome these two challenges, the possibility of adding BCG to the niosomal suspension after formulating and optimising the niosomes has been investigated. This will preserve the drug and reduce the degree of degradation. In formulation F6, BCG was added to the formulation after it was sonicated. Then, HCl was added to start active loading. The results are summarised in Table 5 below.

As shown in Table 5, the %EE of BCG in F6 is similar to that of F3 after sonication (68.59% vs. 67.86%, respectively, after 15 min of drug loading), confirming that loading the drug after optimising the formulation does not compromise %EE. Thus, the addition of a drug after optimising the niosomal formulation should be considered, as it reduces the risk of damaging the drug during thin film hydration.

#### 3.3.7. Main Surfactants Span 60 vs. Span 40

Span 40 is a surfactant that has a structure similar to Span 60 but has a hydrophobic tail that is shorter than Span 60 (15-carbon tail vs. 17-carbon tail, respectively; Figure 10), a lower phase transition temperature (42 °C vs. 53 °C) [35] and smaller molar mass (402.57 g/mol vs. 430.62 g/mol) [31,36].

The effect of the difference in physical properties between Span 40 and Span 60 on entrapment efficiency and niosome size during pH gradient loading of BCG has been studied. Similar to formulation F6, F7 had BCG added to the formulation after it was sonicated. Then, HCl was added to start active loading. The results are summarised in Table 5.

By comparing F7 and F6, it can be concluded that Span 60 niosomes have a better ability to entrap BCG via pH gradient than Span 40 niosomes. This can be explained by learning from liposomes that lipids with a high phase-transition temperature (Tm) have a better ability to maintain a pH gradient [37]. It seems like the same principle applies to surfactants and niosomes. In addition, Span 60 has a longer hydrophobic tail, which can form a thicker and stronger bilayer membrane, resulting in a more substantial barrier and maintaining the pH gradient for a longer period. Niosomes that can maintain a pH gradient for a longer period will result in a better %EE, as the pH gradient is the driving force for entrapping BCG. Similar to liposomes, it appears that when niosomes lose their pH gradient, the %EE will significantly reduce [29].

#### 3.3.8. Effect of Different Buffers on BCG Entrapment Efficiency

- Changing acid buffer

The use of a weak acid instead of HCl has also been studied to assess if reducing the suspension pH using a weak acid can form a more stable pH gradient and thus enhance entrapment efficiency. For this, a citric acid solution (0.25 M) was used, and its effect was studied on both Span 40 and Span 60 formulations. Formulations F8 and F9 were formulated precisely the same way as formulations F6 and F7, respectively. However, instead of adding 0.1 mL of 1 M HCl, 0.5 mL of 0.25 M citric acid was added to reduce the pH of the external medium to 3.2. The results are summarised in Table 5.

The addition of citric acid has resulted in the formation of a green colour, as shown in Figure 11b. However, the colour 15 min after adding HCl was greener than when citric acid was added, indicating a better %EE when HCl was added. This shows that HCl has a better ability to form a pH gradient than citric acid.

However, it is worth noting that citric acid maintained the pH gradient for a longer period than HCl. As shown in Figure 11c, after 60 min of adding either HCl or citric acid, niosomes with HCl lost their pH gradient and turned completely yellow, while niosomes with citric acid maintained the green colour. The colour change also matches the %EE results in Table 5, where the %EE in niosomes with HCl was high after 15 min. Still, they significantly decreased after 1 h (*p* < 0.05), whereas the opposite occurred with niosomes containing citric acid, where %EE was higher after 1 h than after 15 min.

This can be explained by the fact that citric acid has a buffering effect, and it releases H^+^ ions more slowly than HCl, which is a strong acid that dissociates completely in water [38,39]. Also, citric acid is very water soluble and has pKa values of 3.15, 4.78 and 6.40 for its three carboxylic acids [38]. At pH 3.5, which is the pH of the external media after citric acid addition, citric acid will mainly be in its ionised form [38]. Given that citric acid is mainly ionised and water soluble, it will not cross the niosomes bilayer membrane easily and will mostly remain in the external media. However, HCl creates a stronger initial pH gradient, which allows for a quicker influx of BCG, resulting in a higher %EE after 15 min. Thus, the use of citric acid at 0.25 M concentration is not recommended over the use of HCl for loading BCG.

Another theoretical advantage of using HCl instead of citric acid is that each citric acid molecule has three carboxylic acid functional groups, and Span surfactants have three secondary alcohol functional groups (Figure 10) that can react with citric acid to form esters (esterification reaction) [38]. Cholesterol and Solutol HS-15 also have alcohol functional groups that can react with citric acid carboxylic acids (Figure 10). The effect of citric acid on the stability of pH gradient niosomes containing Span 60, cholesterol and Solutol HS-15 is unknown. Also, the toxicity of the new products formed by esterification reactions is unknown.

Moreover, loaded drugs that contain alcohol or amine functional groups can also form esters with citric acid. This change in the drug structure can affect entrapment efficiency and prevent the drug from binding effectively to its final target. Also, the toxicity of the new product will be unknown.

b. Changing alkaline buffer

Changing the alkaline buffer has also been studied. HEPES buffer (0.1 M, pH 8.0) was used instead of Trizma buffer in formulations F10 and F11. HCl (0.1 mL of 1 M) was added to 1 mL of F10, while citric acid (0.5 mL of 0.25 M) was added to 1 mL of F11 to induce a pH gradient and reduce the external pH to 2.8 or 3.36, respectively. Results are summarised in Table 6 below.

**Figure 12:**
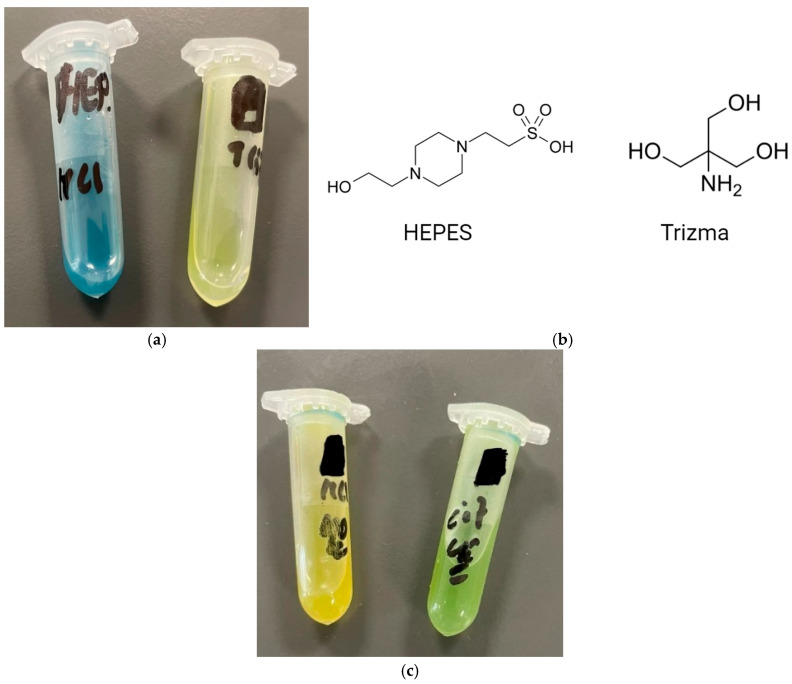
(**a**) Left Eppendorf shows F10 (made in HEPES buffer) after 1 h of adding HCl. Right Eppendorf shows F6 (made in Trizma buffer) after 1 h of adding HCl. The %EE of F10 after 1 h of drug loading is 72.85%, while that of F6 after 1 h of drug loading is 34.61%. (**b**) Structure of HEPES and Trizma compounds. (**c**) Formulation F6 at 40 °C after 10 min of adding HCl (left Eppendorf) and F8 at 40 °C after 10 min of adding citric acid (right Eppendorf). The %EE of F6 after 10 min of drug loading at 40 °C is 11.19%, while that of F8 after 10 min of drug loading at 40 °C is 40.44%.

Unlike the Trizma buffer, the HEPES buffer showed better entrapment efficiency after 1 h with both HCl and citric acid than after 15 min. Moreover, as shown in Figure 12a below, HEPES has better abilities to control and maintain a pH gradient for a longer time, as it retains its blue colour for 1 h, whereas similar niosomes prepared in the Trizma buffer turn yellow after 1 h, indicating that the pH gradient had been lost.

To understand the reasons behind this observation, the structural and physical properties of Trizma and HEPES compounds should be compared. Figure 12b above shows the structure of HEPES vs. Trizma compounds. Table 7 below compares the properties of Trizma and HEPES compounds.

It can be concluded from Table 7 below that HEPES is more hydrophilic than Trizma, where it has higher water solubility (70.36 g/100 mL vs. 67.8 g/100 mL, respectively) and Log Pow of HEPES is more negative than Trizma (−3.85 vs. −2.31, respectively). Also, since HEPES is larger than Trizma with a molar mass of almost double (238.30 g/mol vs. 121.14 g/mol), it is more difficult for HEPES than Trizma to cross the niosomal bilayer membrane. Moreover, at the pH this study is being conducted (eight and nine inside niosomes for HEPES and Trizma, respectively, with <3.5 outside the niosomes), HEPES will be more ionised inside the niosome core than Trizma (~50% vs. ~10%) and less ionised outside niosomes than Trizma (~50% vs. 100%). During the pH gradient, Trizma will be encouraged to leave the niosomes to become ionised in the acidic media surrounding the niosomes, while the HEPES percentage of ionisation inside and outside the niosomes will be the same. Thus, the driving force for it to leave the niosomes is weaker than that of Trizma. When the buffer leaves the niosomes, the niosomes become more susceptible to substantial pH changes due to the added acid, and thus, they can lose their pH gradient.

As a result, since it is more difficult for HEPES to cross the bilayer membrane and the pH gradient does not encourage it to leave the niosomes as much as the case with Trizma, HEPES can maintain the pH gradient for a longer time than Trizma.

#### 3.3.9. Effect of Temperature on BCG Loading

In liposomes, raising the temperature of the suspension above the phase transition temperature of the main lipid during drug loading by pH gradient can increase the entrapment efficiency by adjusting the fluidity of the bilayer membrane [43]. This technique has been utilised in liposomes, and its influence on the niosomes’ membrane and the ability to maintain a pH gradient have been studied in this research.

Formulations F6, F7, F8 and F9 were loaded with BCG using a pH gradient initiated by either HCl (F6 and F7) or citric acid (F8 and F9) at 40 °C to study the effect of raising the temperature on %EE. Entrapment efficiency was measured after 10 min and 40 min. The 10 min and 40 min timepoints were chosen based on colour observations, where after 10 min, the niosomal suspension starts to turn yellow. The results are summarised in Table 8.

It can be concluded from Table 8 that increasing the temperature can significantly reduce the %EE in the pH gradient loading of niosomes. For formulation F6, the maximum %EE was much higher when loading was conducted at room temperature than at 40 °C (68.59% vs. 11.19%, *p* < 0.05). The same outcome was observed for the F7 formulation (53.3% vs. 19.59%, *p* < 0.05). This is because an increasing temperature makes the niosomal bilayer more permeable, and as a result, niosomes lose their pH gradient. This is evident in Figure 12c, where the pH gradient was lost after just 10 min of adding HCl at a temperature of 40 °C. Comparing this to Figure 11b, the pH gradient was not lost after 15 min when loading was conducted at room temperature. This indicates that, unlike active loading in many liposomal formulations, the niosomal formulation containing Span 60, Solutol HS-15 and cholesterol does not require a high drug loading temperature, and room temperature can be sufficient for the active loading of drugs.

Raising the temperature is known to increase the kinetic energy of the molecules and increase the permeability of biological membranes. It can be concluded that raising the temperature can also encourage Trizma to leave the niosomes through the bilayer membrane and thus promote the loss of pH gradient, which can result in a lower %EE [43].

However, it should be noted that in pH gradient loading induced by citric acid, increasing the temperature to 40 °C has achieved a higher maximum %EE at a shorter time when compared to loading at RT, where for formulation F8, %EE after 10 min at 40 °C was 40.44% compared to 29.63% after 60 min at RT (Table 6 and Table 8). However, it should be noted that at room temperature, the pH gradient lasted for a longer time than at 40 °C, where at room temperature, %EE increased from 24.18% at 15 min to 29.63% at 60 min, while at 40 °C, %EE decreased from 40.44% at 10 min to 23.56 at 40 min. This indicates that when using a weak acid, increasing the temperature can quickly achieve a higher %EE; however, the pH gradient can also be lost more quickly compared to loading at RT. It can also be concluded that using a weak acid can maintain a pH gradient for a longer time at 40 °C than using a strong acid (Figure 12c).

#### 3.3.10. Effect of Different Ratios of Cholesterol to Span 60 on Active Loading of BCG and Its %EE

Cholesterol affects the permeability and fluidity of bilayer membranes [43]. Thus, its concentration in the formulation can influence membrane permeability and, consequently, the %EE.

The effect of changing the Span 60 to the cholesterol molar ratio was studied in this research. Formulations F12 and F13 have different ratios of cholesterol to Span 60, as summarised in Table 1. The results of active loading are summarised in Table 9 below.

It can be concluded that reducing the cholesterol to Span 60 molar ratio from 45:45 to 35:55 (F12) did not affect the entrapment efficiency of BCG after 15 min (68.59% vs. 66.52%, respectively). However, it reduced the leakage of the drug over time, where after 60 min of the niosomes’ exposure to the pH gradient, the %EE of BCG was 34.61% vs. 53.11%, respectively.

However, increasing the cholesterol to Span 60 ratio from 45:45 to 55:35 had the opposite effect, where, after 15 min, the %EE achieved was 68.59% vs. 59.77%, respectively. After 60 min of niosomal exposure to a pH gradient, the drug leaked more, with %EE being 34.61 vs. 27.06, respectively.

Cholesterol is known to have a significant effect on the rigidity, fluidity, and permeability of the niosomal membrane, and %EE [1,44]. This study has confirmed that increasing the cholesterol molar ratio to 55% in the formulation made the membrane leakier, as it reduced the %EE and reduced the membrane’s ability to maintain a pH gradient.

#### 3.3.11. Concluding Remarks on the Use of Bromocresol Green in pH Gradient Studies

Using BCG to study pH gradients in niosomes has proven to be a valuable tool for understanding these pH gradients. The ability of BCG to change colour when exposed to different pH environments and its ability to be actively loaded into niosomes have enabled the study of how different parameters can affect active drug loading and the stability of the pH gradient in niosomes.

Through this tool, it was possible to visualise and study how the following factors can influence the stability of pH gradient and active drug loading in niosomes: the type of surfactant used (Span 60 vs. Span 40), the choice of co-surfactant (Solutol HS-15, Cremophor RH 40, or Cremophor ELP), the composition of both alkaline and acidic buffers, the loading temperature and duration, the morphology of the niosomes (MLV vs. LUV) and the cholesterol content within the formulation.

These discoveries will aid in understanding how to successfully formulate pH gradient niosomes with therapeutic drugs and the effect of different formulation compositions and buffer choices on enhancing the active loading of therapeutic drugs into niosomes. In this research, these findings will be applied to improve the entrapment efficiency of the therapeutic agent doxorubicin into niosomes, as will be discussed in Section 3.4. The findings will not be limited to doxorubicin but rather can be applied to enhance the entrapment efficiency of various drugs that can be actively loaded using a pH gradient.

### 3.4. Studying the Entrapment of Doxorubicin Using pH Gradient

**Figure 13:**
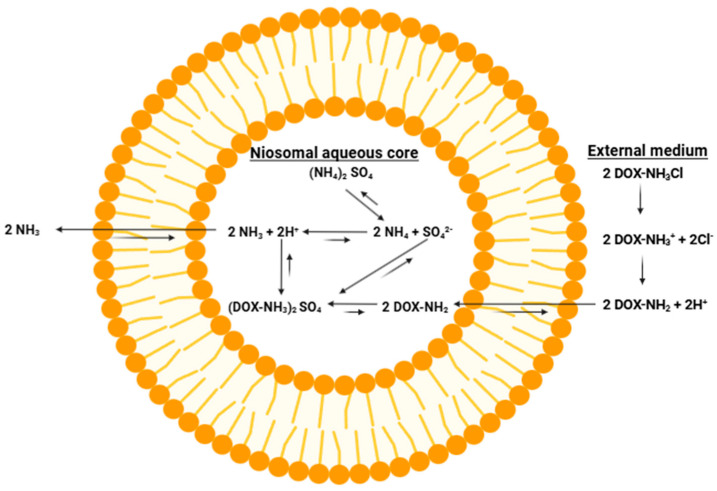
Concentration of ammonium sulfate in the niosomal core is much higher than that outside the niosomes. DOX-NH_3_Cl is doxorubicin hydrochloride. Doxorubicin will become de-ionised in the external medium and cross the niosomal membrane. In the niosomal aqueous core, doxorubicin amine will receive one hydrogen from ammonia and become positively charged. Two of the positively charged doxorubicin molecules will form a complex with the negatively charged sulfate (SO_4_^2−^) ion. This complex is not soluble, and doxorubicin will precipitate within the niosomes, allowing more doxorubicin to enter the niosomal core. In exchange for doxorubicin entrance, ammonia will leave the niosomes through the niosomal membrane as it becomes neutral after donating its hydrogen to doxorubicin amine [45] (created using BioRender).

After demonstrating the ability of niosomes containing Span 60, cholesterol and Solutol HS-15 to maintain a pH gradient, the ability of this niosomal formulation to entrap an actual drug using salt and pH gradients has also been investigated. For this purpose, doxorubicin, a model drug commonly loaded into liposomes using pH and salt gradients, has been employed. A common method for loading doxorubicin into liposomes involves using an ammonium sulfate gradient, where liposomes are prepared in an ammonium sulfate solution, followed by a buffer exchange to remove ammonium sulfate from the outside of the liposomes. Thus, the concentration of ammonium sulfate is much higher in the liposomal core than outside the liposomes. When an ammonium sulfate gradient is formed, neither the ammonia nor the sulfate can leave the liposomal core because they are ions and therefore cannot cross the liposomal membrane [45]. The ability of niosomes containing Span 60, cholesterol and Solutol HS-15 to form and maintain an ammonium sulfate gradient has been tested using doxorubicin. Figure 13 shows the mechanism of entrapping doxorubicin inside niosomes using an ammonium sulfate gradient.

The most optimised formulation from BCG experiments was one containing Span 60, cholesterol and Solutol HS-15 at a molar ratio of 45:45:10. Solutol HS-15 is a suitable candidate for use in this formulation, as it is safe to use in injectables. Solutol HS-15 was also found to inhibit P-gp protein in cancer cells, resulting in a higher accumulation and toxicity of anticancer drugs. It also exhibits low haemolytic properties, indicating that it has minimal irritation and toxicity to healthy cells [20,21].

#### 3.4.1. Passive Loading of Doxorubicin into Niosomes

Doxorubicin is a cytotoxic drug that is administered intravenously, and the niosomes used to load doxorubicin should be small in size. For this reason, loading doxorubicin during the hydration of the thin film is not considered, as the size produced after hydration is high (>1000 nm), as previously established (Table 3). Reducing the niosomes’ size using extrusion or sonication can result in drug loss and leakage, as previously discussed. For this reason and comparison purposes, passive loading in formulation F1-D was considered by adding doxorubicin to niosomes prepared with Trizma buffer only (without a pH gradient). The %EE achieved after 1 h loading was 36.42% (±1.01).

#### 3.4.2. pH Gradient Loading of Doxorubicin into Niosomes

An ammonium sulfate gradient will be established to actively load doxorubicin into niosomes by preparing the niosomes in ammonium sulfate and then resuspending them in PBS (formulation F2-D) or Trizma (formulation F3-D). Drug loading was conducted at RT and 60 °C. The results are summarised in Table 10.

In ammonium sulfate gradient loading of doxorubicin through liposomes, the temperature is usually raised above the phase transition temperature of the main lipid in the formulation to facilitate the permeability of doxorubicin through the membrane. This ensures the high entrapment efficiency of doxorubicin and a high drug-to-lipid molar ratio.

In niosomes, the opposite is obtained. Raising the temperature to 60 °C resulted in lower entrapment efficiency when using PBS and Trizma as external buffers. This can be explained using the results in Section 3.3.9, where raising the temperature in niosomes resulted in losing the pH gradient and reducing the %EE. Using Trizma as an external buffer has achieved a better %EE than PBS after 1 h of drug loading (57.14% vs. 28.07% at RT). This can be because Trizma can maintain a pH gradient more efficiently than PBS in niosomes.

#### 3.4.3. Effect of Ammonium Sulfate Concentration

Some literature has used 120 mM ammonium sulfate buffer in the inner core of liposomes, while other literature has used a 250 mM concentration [46,47]. The efficiency of a 250 mM ammonium sulfate concentration in niosomes was tested in formulation F4-D (refer to Table 2). Drug loading was conducted at RT and 60 °C. The results are summarised in Table 11.

Using 250 mM ammonium sulfate instead of 120 mM did not result in an increase in the maximum %EE at either temperature. However, there is a size increase in niosomes after 1 h of drug loading, where at RT the niosomes’ size was increased from 389.1 nm to 543.6 nm. This was not observed when the ammonium sulfate concentration was 120 mM, as the size did not change significantly after 1 h of drug loading at room temperature (360 nm before drug loading vs. 367.3 nm after drug loading). This indicates that using 120 mM ammonium sulfate is preferred over the 250 mM concentration, as it reduces the amount of ammonium sulfate needed, improves the size after loading and results in a similar maximum %EE.

#### 3.4.4. Effect of Percentage of Cholesterol in the Formulation

As previously discussed in Section 3.3.10, the amount of cholesterol in the formulation can play a crucial role in membrane stability and drug loading. A new formulation (F5-D) was tested after reducing % of cholesterol to 35%. Additionally, the effect of mixing the formulation using a magnetic bar and a magnetic stirrer during drug loading to enhance the %EE was studied. The size and %EE results of active loading are summarised in Table 12.

Reducing the cholesterol content to 35% resulted in a higher entrapment efficiency compared to F4-D, which contained 45% cholesterol (64.93% vs. 56.47%, respectively). It also increased the size of niosomes after sonication to 473.5 nm, compared to 389.1 nm in formulation F4-D. However, the size remained stable during drug loading (473.5 nm before drug loading vs. 464.8 nm after 1 h of drug loading). This suggests that reducing the cholesterol concentration to 35% can increase the %EE. Size can be managed using extrusion, as will be discussed in the next section (Section 3.4.5). Mixing using a magnetic bar and a magnetic stirrer was also tested. The effect of mixing on entrapment efficiency and size is reported in Table 12.

It can be concluded that mixing did not improve the %EE. It also had no significant effect on the niosomal size. For this reason, it is not essential to apply mixing during the loading of doxorubicin in niosomes.

#### 3.4.5. Effect of Extrusion on Size and %EE

Extrusion is a common method that is applied to liposomes and niosomes to reduce their size [1,48]. Extrusion includes passing the niosomes through a membrane that has pores of a specific size [1]. Through pressure, niosomes are forced through the membrane, and as they pass through the tiny pores (100 nm), their size is reduced.

Formulation F5-D was selected for extrusion because it exhibited the highest entrapment efficiency. The extruded formulation (F5-D-E) is summarised in Table 1. The size and %EE of the F5-D-E formulation are shown in Table 13.

Sonication is an effective technique for reducing the size of niosomes. However, the formulation PDI might remain high, as shown in Table 10, Table 11 and Table 12. Hence, extrusion was applied as per Table 13, extruding the niosomes successfully reduced the size to 241.1 nm and improved the PDI of the formulation. It also increased the %EE to 68.28%. The niosomes’ size remained stable after 1 h of drug loading, with a slight increase in the particles’ PDI (Table 13).

Formulating doxorubicin niosomes through this feasibility study, incorporating Solutol HS-15, which can inhibit P-gp in cancer cells, may lead to an increase in the accumulation of doxorubicin in cancer cells, providing a cost-effective option for cancer treatment.

## 4. Conclusions and Future Perspective

In niosomes, co-surfactants are essential for establishing and maintaining a pH gradient. Without co-surfactants, niosomes will collapse when exposed to acidic media. Different co-surfactants have varying abilities to support niosomes in forming and maintaining pH gradients, resulting in different entrapment efficiencies. Moreover, different buffers have varying effects on drug loading and influence the duration during which the pH gradient is maintained. The active loading of BCG and doxorubicin was successful in niosomes containing Solutol HS-15 as a co-surfactant. Solutol HS-15 containing niosomes showed the highest BCG EE (67.86%) and doxorubicin EE (68.28%).

To our knowledge, niosomes containing Solutol HS-15 have not been used to entrap cytotoxic drugs. However, the formulation requires more optimisation to enhance the %EE of doxorubicin. Different ammonium salts can be tried to improve the %EE. It was found that ammonium citrate had a better %EE of doxorubicin in liposomes than ammonium sulfate (100% vs. 95%) [49]. Additionally, the external buffer can be adjusted to investigate its effect on %EE. For example, this research found that HEPES buffer can maintain a pH gradient for a longer time than Trizma. Using HEPES might result in better doxorubicin %EE.

Additionally, the concentration of doxorubicin can be increased to more than 0.1 mg/mL by lyophilisation. Lyophilisation can also result in a very stable formulation. Lyophilisation can be used to concentrate and stabilise the formulation.

Solutol HS-15 is promising as it can be used to enhance the cytotoxicity of doxorubicin by inhibiting P-gp [20,21]. The cytotoxicity of the optimised formulation should be tested in the future on cancer cells and in animal trials. Also, in vitro drug release and in vivo pharmacokinetics should be studied.

## Figures and Tables

**Table 1:** Niosomal formulations for entrapping bromocresol green (BCG) via pH gradient.

Formulation Code	Co-Surfactant Used	Span Surfactant Used	Total Number of Moles of Ingredients (µmol)	Molar Ratio of Span Surfactant: Cholesterol: Co-Surfactant	Thin Film Hydration Buffer
F1	Cremophor RH40	Span 60	60	45:45:10	5 mL of Trizma (0.1 M, pH 9.0, 0.1 mg/mL BCG)
F2 *	Cremophor ELP	Span 60	60	46.5:46.5:7.0	5 mL of Trizma (0.1 M, pH 9.0, 0.1 mg/mL BCG)
F3	Solutol HS-15	Span 60	60	45:45:10	5 mL of Trizma (0.1 M, pH 9.0, 0.1 mg/mL BCG)
F4	N/A	Span 60	60	50:50:0	5 mL of Trizma (0.1 M, pH 9.0, 0.1 mg/mL BCG)
F5	N/A	Span 40	60	50:50:0	5 mL of Trizma (0.1 M, pH 9.0, 0.1 mg/mL BCG)
F6	Solutol HS-15	Span 60	60	45:45:10	4.5 mL of Trizma (0.1 M, pH 9.0)
F7	Solutol HS-15	Span 40	60	45:45:10	4.5 mL of Trizma (0.1 M, pH 9.0)
F8	Solutol HS-15	Span 60	60	45:45:10	4.5 mL of Trizma (0.1 M, pH 9.0)
F9	Solutol HS-15	Span 40	60	45:45:10	4.5 mL of Trizma (0.1 M, pH 9.0)
F10	Solutol HS-15	Span 60	60	45:45:10	4.5 mL of HEPES (0.1 M, pH 8.0)
F11	Solutol HS-15	Span 60	60	45:45:10	4.5 mL of HEPES (0.1 M, pH 8.0)
F12	Solutol HS-15	Span 60	60	35:55:10	4.5 mL of Trizma (0.1 M, pH 9.0)
F13	Solutol HS-15	Span 60	60	55:35:10	4.5 mL of Trizma (0.1 M, pH 9.0)

* In formulation F2, the ratio of 45:45:10 of Span 60–cholesterol–Cremophor ELP was trialled. Still, it did not produce large unilamellar vesicle (LUV) niosomes after sonication, which is one of the targets for these formulations to study the effect of size on %EE. The ratio shown in this table (46.5:46.5:7.0) was tested and produced LUV niosomes upon sonication. For this reason, it was chosen.

**Table 2:** Niosomal formulations for entrapping doxorubicin via pH gradient.

Formulation Code	Molar Ratio of Span 60: Cholesterol: Solutol HS-15	Total Number of Moles of Ingredients (µmol)	Hydration Buffer	External Buffer Exchange Method
F1-D	45:45:10	120	5 mL of Trizma (0.1 M, pH 9.0)	N/A
F2-D	45:45:10	120	5 mL Ammonium sulfate (0.12 M)	Sephadex G50 (in PBS 1×)
F3-D	45:45:10	120	5 mL Ammonium sulfate (0.12 M)	Sephadex G50 (in Trizma 0.1 M)
F4-D	45:45:10	120	5 mL Ammonium sulfate (0.25 M)	Sephadex G50 (in Trizma 0.1 M)
F5-D	55:35:10	120	5 mL Ammonium sulfate (0.12 M)	Sephadex G50 (in Trizma 0.1 M)
F5-D-E *	55:35:10	120	5 mL Ammonium sulfate (0.12 M)	Sephadex G50 (in Trizma 0.1 M)

* Niosomal formulation F5-D-E was extruded after sonication.

**Table 3:** Size and %EE (n = 3) before and after sonication for passively loaded BCG in three formulations, F1–F3. All three formulations consist of Span 60 and cholesterol but contain different co-surfactants: Cremophor RH40 (F1), Cremophor ELP (F2) and Solutol HS-15 (F3).

Formulation	Before Sonication			After Sonication		
	%EE (±SD)	Niosomes Size– d nm (±SD)	PDI (±SD)	%EE (±SD)	Niosomes Size– d nm (±SD)	PDI (±SD)
F1	3.92 ± 0.07	1631 ± 191.0	0.78 ± 0.28	2.37 ± 0.86	177.9 ± 8.28	0.52 ± 0.07
F2	4.30 ± 1.34	1687 ± 493.8	0.72 ± 0.18	2.55 ± 1.77	219.1 ± 39.38	0.46 ± 0.06
F3	4.99 ± 1.30	1171 ± 119.1	0.76 ± 0.09	0.00	159.6 ± 4.34	0.53 ± 0.03

**Table 4:** Effect of size on entrapment efficiency (n = 3) using pH gradient. The %EE in the three niosomal formulations (F1–F3) was measured before and after their size reduction by sonication to study the effect of size on pH gradient active drug loading.

Formulation	Before Sonication				After Sonication			
	Niosomes Size– d nm (±SD)	PDI (±SD)	%EE		Niosomes Size– d nm (±SD)	PDI (±SD)	%EE	
			15 min * (±SD)	60 min ** (±SD)			15 min * (±SD)	60 min ** (±SD)
F1	1631 ± 191.0	0.78 ± 0.28	55.73 ± 1.15	61.80 ± 1.53	177.9 ± 8.28	0.52 ± 0.07	15.57 ± 1.72	15.09 ± 2.74
F2	1687 ± 493.8	0.72 ± 0.18	41.45 ± 1.68	39.83 ± 1.74	219.1 ± 39.38	0.46 ±0.06	17.81 ± 4.48	13.90 ± 2.58
F3	1171 ± 119.1	0.76 ± 0.09	57.45 ± 1.97	49.47 ± 2.52	159.6 ± 4.34	0.53 ± 0.03	67.86 ± 4.26	42.02 ± 4.81

* %EE measured after 15 min of adding HCl. ** %EE measured after 60 min of adding HCl

**Table 5:** Measuring noisome size, PDI and %EE of BCG in four niosomal formulations (F6–F9), (n = 3). BCG was added after the niosomes were formulated and sonicated. Formulations F6 and F8 contain Span 60, while formulations F7 and F9 contain Span 40. pH gradient was achieved in formulations F6 and F7 by the addition of HCl, while in formulations F8 and F9, it was achieved by adding citric acid.

Formulation Code	Niosomes Size– d nm (±SD)	PDI (±SD)	%EE	
			15 min * (±SD)	60 min ** (±SD)
F6	147.8 (±6.30)	0.39 (±0.04)	68.59 (±0.70)	34.61 (±2.97)
F7	226.9 (±16.08)	0.37 (±0.04)	53.30 (±2.88)	28.53 (±7.91)
F8	147.8 (±6.30)	0.39 (±0.04)	24.18 (±5.56)	29.63 (±3.95)
F9	226.9 (±16.08)	0.37 (±0.04)	14.86 (±0.56)	24.34 (±3.04)

* %EE measured after 15 min of adding the acid. ** %EE measured after 60 min of adding the acid

**Table 6:** F10 and F11 size, PDI and %EE using pH gradient (n = 3). F10 and F11 were prepared in HEPES buffer (0.1 M, pH 8.0) instead of Trizma and were sonicated to reduce their size. A pH gradient was created by either adding HCl (F10) or citric acid (F11) to reduce the external pH.

Formulation	Niosomes Size– d nm (±SD)	PDI (±SD)	%EE (for HCl)		%EE (for Citric Acid)	
			15 min * (±SD)	60 min ** (±SD)	15 min * (±SD)	60 min ** (±SD)
F10	283.4 ± 34.08	0.38 ± 0.07	53.19 ± 2.90	72.85 ± 2.72	-	-
F11	283.4 ± 34.08	0.38 ± 0.07	-	-	25.92 ± 1.29	31.08 ± 1.27

* %EE measured after 15 min of adding HCl/citric acid. ** %EE measured after 60 min of adding HCl/citric acid

**Table 7:** Comparison between Trizma’s and HEPES’ physical properties [40,41,42].

Physical Property	HEPES	Trizma
Molar mass	238.30 g/mol	121.14 g/mol
Water solubility	70.36 g/100 mL	67.80 g/100 mL
Number of ionisable functional groups	3	1
pKa of base	7.5 and 3.0	8.1
pKa of acid	Always ionised	N/A
Ionisation state at pH > 5.4	At pH 8 > 50% of HEPES will be negatively charged, and the other 50% will be zwitterionic (neutral)	At pH 9, only ~10% will be ionised
Ionisation state at pH < 3.8	At pH 2.8–3.36 (i.e., after adding HCl or Citric acid), around 50% of HEPES will be positively charged, and the other 50% will be zwitterionic (neutral)	At pH < 3.8, 100% will be ionised
Partition coefficient Octanol-water (Log Pow)	−3.85	−2.31

**Table 8:** Results of size, PDI and %EE (n = 3) of niosomes loaded via pH gradient at 40 °C. This table shows the results of %EE when drug loading takes place at elevated temperature, 40 °C.

Formulation	Niosomes Size– d nm (±SD)	PDI (±SD)	%EE (for HCl) at 40 °C		%EE (for Citric Acid) at 40 °C	
			10 min * (±SD)	40 min ** (±SD)	10 min * (±SD)	40 min ** (±SD)
F6	147.8 ± 6.30	0.39 ± 0.04	11.19 ± 2.49	2.46 ± 1.0	-	-
F7	226.9 ± 16.08	0.37 ± 0.04	19.59 ± 6.77	4.52 ± 5.34	-	-
F8	147.8 ± 6.30	0.39 ± 0.04	-	-	40.44 ± 7.79	23.56 ± 1.62
F9	226.90 ± 16.08	0.37 ± 0.04	-	-	26.60 ± 1.42	21.00 ± 1.10

* %EE measured after 10 min of adding HCl/citric acid. ** %EE measured after 40 min of adding HCl/citric acid.

**Table 9:** Size, PDI and %EE (n = 3) of formulations containing different molar ratios of Span 60 to cholesterol. The molar ratio of Span 60 to cholesterol to Solutol HS-15 in formulation F12 is 35:55:10, whereas in F13, it is 55:35:10. The size was measured both before and after sonication, but the drug loading was performed using sonicated niosomes.

Formulation	Before Sonication		After Sonication		%EE (for HCl) After Sonication	
	Niosomes Size– d nm (±SD)	PDI (±SD)	Niosomes Size– d nm (±SD)	PDI (±SD)	15 min * (±SD)	60 min ** (±SD)
F12	1394 ± 68.25	0.60 ± 0.12	320.9 ± 20.64	0.53 ± 0.10	66.52 ± 1.4	53.11 ± 4.86
F13	1390 ± 87.54	0.45 ± 0.16	148.4 ± 9.65	0.35 ± 0.07	59.77 ± 3.46	27.06 ± 6.50

* %EE measured after 15 min of adding HCl. ** %EE measured after 60 min of adding HCl.

**Table 10:** Size, PDI and %EE (n = 2) of doxorubicin loaded at room temperature (RT) and at 60 °C. The core of niosomes contained ammonium sulfate (120 mM), while the external medium consisted of either PBS (1X, pH 7.4) in formulation F2-D or Trizma buffer (0.1 M, pH 9.0) in formulation F3-D.

Formulation	Niosomes’ Size After Sonication and Before Entrapment d nm (±SD)	PDI	Measuring Size and %EE at RT				Measuring Size and %EE at 60 °C			
			%EE (After 30 min) (±SD)	%EE (After 60 min) (±SD)	Niosomes Size– d nm (±SD) (After 60 min)	PDI (After 60 min) (±SD)	%EE (After 30 min) (±SD)	%EE (After 60 min) (±SD)	Niosomes Size– d nm (±SD) (After 60 min)	PDI (After 60 min) (±SD)
F2-D	384.6 ± 10.8	0.45 ± 0.08	28.07% ± 0.57	28.07 ± 0.57	389.2 ±48.3	0.35 ± 0.03	21.55 ± 2.41	22.33 ± 2.40	413.0 ± 31.82	0.36 (±0.05)
F3-D	360.0 ± 2.54	0.44 ± 0.02	55.44 ± 2.41	57.14 ± 3.85	367.3 ±18.8	0.46 ± 0.02	26.53 ± 3.85	32.31 ± 4.33	380.8 ± 10.59	0.37 (±0.04)

**Table 11:** Size, PDI and entrapment efficiency (n = 2) of niosomal formulation prepared in 250 mM ammonium sulfate buffer, and the drug was loaded at RT and 60 °C. The niosomal external buffer consisted of Trizma buffer (0.1 M, pH 9.0).

Formulation	Niosomes’ Size After Sonication and Before Entrapment d nm (±SD)	PDI	Measuring Size and %EE at RT				Measuring Size and %EE at 60 °C			
			%EE (After 30 min) (±SD)	%EE (After 60 min) (±SD)	Niosomes Size– d nm (±SD) (After 60 min)	PDI (After 60 min) (±SD)	%EE (After 30 min) (±SD)	%EE (After 60 min) (±SD)	Niosomes Size– d nm (±SD) (After 60 min)	PDI (After 60 min) (±SD)
F4-D	389.1 (±30.26)	0.51 ± 0.04	56.76 ± 0.42	56.47 ± 0	543.6 ± 8.752	0.44 ± 0.06	25.96 ± 3.44	27.56 ± 2.98	693.1 ± 29.68	0.45 (±0.05)

**Table 12:** Size, PDI and %EE (n = 2) of formulation F5-D prepared with a niosome core containing 120 mM ammonium sulfate and external buffer was Trizma (0.1 M, pH 9.0). F5-D contains the molar ratio of Span 60 to cholesterol to Solutol HS-15 of 55:35:10. Drug loading was performed either accompanied by mixing or without mixing as an additional driving force for drug loading.

Formulation	Niosomes’ Size After Sonication and Before Entrapment d nm (±SD)	PDI	Without Mixing				With Mixing			
			%EE (After 30 min) (±SD)	%EE (After 60 min) (±SD)	Niosomes Size– d nm (±SD) (After 60 min)	PDI (After 60 min) (±SD)	%EE (After 30 min) (±SD)	%EE (After 60 min) (±SD)	Niosomes Size– d nm (±SD) (After 60 min)	PDI (After 60 min) (±SD)
F5-D	473.5 ± 37.80	0.48 ± 0.08	64.93 ± 5.28	64.93 ± 5.28	464.8 ± 82.76	0.54 ± 0.09	62.69 ± 2.11	63.43 ± 3.17	456.9 ± 19.9	0.50 ± 0.03

**Table 13:** The size, PDI and entrapment efficiency (n = 2) of the F5-D-E, which is similar to formulation F5-D in composition, but it was extruded to reduce and unify the size. Drug loading was performed for 1 h at RT, and the size was measured after completion of drug loading as well.

Formulation	Niosomes’ Size After Extrusion d nm (±SD)	PDI	%EE After 60 min	Niosomes Size– d nm (±SD) (After 60 min)	PDI After 60 min
F5-D-E	241.1 ± 1.65	0.18 ± 0.03	68.28 ± 0.53	265.8 ± 10.05	0.22 ± 0.02

## Data Availability

Data is contained within the article.
